# Supplementary material for: Comparison of Kinetic Models Applied for Transport Description in Polymer Inclusion Membranes
Source: Membranes (Basel). 2023 Feb 16;13(2):236. doi: 10.3390/membranes13020236 (PMC9967031; doi:10.3390/membranes13020236)
Supplement: Supplementary file 1 [file membranes-13-00236-s001.zip › membranes-2141890-supplementary.pdf]

Table. S1. List of differential equations used in an appropriate models.

Model No.	Scheme	V=f(t)	Differential equations
1	A→B	NO	$\frac{dc_f}{dt} = -k_1 c_f$<br>$\frac{dc_s}{dt} = \frac{V_f}{V_s} k_1 c_f$
1a	A→B	YES	$\frac{dc_f}{dt} = -k_1 c_f$<br>$\frac{dc_s}{dt} = \frac{V_f}{V_s} k_1 c_f$
2	A↔B	YES	$\frac{dc_s}{dt} = \frac{V_f}{V_s} k_1 c_f - k_{-1} c_s$<br>$\frac{dc_f}{dt} = -k_1 c_f + \frac{V_s}{V_f} k_{-1} c_s$
3	A→B→C	YES	$\frac{dc_f}{dt} = -k_1 c_f$<br>$\frac{dc_{LM}}{dt} = \frac{V_f}{V_{LM}} k_1 c_f - k_2 c_{LM}$<br>$\frac{dc_s}{dt} = \frac{V_{LM}}{V_s} k_2 c_{LM}$
4	A↔B→C	YES	$\frac{dc_f}{dt} = -k_1 c_f + \frac{V_{LM}}{V_f} k_{-1} c_{LM}$<br>$\frac{dc_{LM}}{dt} = \frac{V_f}{V_{LM}} k_1 c_f - k_{-1} c_{LM} - k_2 c_{LM}$<br>$\frac{dc_s}{dt} = \frac{V_{LM}}{V_s} k_2 c_{LM}$
5	A→B	YES	$\frac{dc_f}{dt} = -k_1$

Table S2. Atomic absorption spectroscopy operating parameters.

Element	Wavelength (nm)	Slit (nm)
Cd(II)	228.8	0.5
Zn(II)	213.9	1.0
Pb(II)	283.3	1.0
Cu(II)	324.8	0.5

Table S3. The calculated kinetic parameters and initial maximum fluxes for the system with TOPO as a carrier.

Ion	No.	Scheme	$V = f(t)$	k_1 [1/cm]	k_{-1} [1/cm]	k_2 [1/cm]	J_M [mol/cm²s]
**Cd(II)**	1	A→B	NO	1.229×10⁻⁵			3.006×10⁻¹⁰
	1a	A→B	YES	1.203×10⁻⁵			2.926×10⁻¹⁰
	2	A↔B	YES	1.202×10⁻⁵	5.030×10⁻¹⁴		2.924×10⁻¹⁰
	3	A→B→C	YES	**1.248×10⁻⁵**		**2.504×10⁻⁴**	**3.036×10⁻¹⁰**
	4	A↔B→C	YES	1.228×10⁻⁵	4.456×10⁻⁹	2.637×10⁻⁴	2.989×10⁻¹⁰
**Zn(II)**	1	A→B	NO	1.437×10⁻⁵			3.400×10⁻¹⁰
	1a	A→B	YES	1.416×10⁻⁵			3.332×10⁻¹⁰
	2	A↔B	YES	1.416×10⁻⁵	1.803×10⁻¹³		3.332×10⁻¹⁰
	3	A→B→C	YES	**1.478×10⁻⁵**		**3.280×10⁻⁴**	**3.477×10⁻¹⁰**
	4	A↔B→C	YES	1.445×10⁻⁵	1.216×10⁻⁸	3.365×10⁻⁴	3.400×10⁻¹⁰
**Pb(II)**	1	A→B	NO	1.020×10⁻⁶			2.411×10⁻¹¹
	1a	A→B	YES	9.953×10⁻⁷			2.342×10⁻¹¹
	2	A↔B	YES	**1.056×10⁻⁶**	**4.270×10⁻⁷**		**2.485×10⁻¹¹**
	3	A→B→C	YES	9.954×10⁻⁷		9.990×10⁻²	2.342×10⁻¹¹
	4	A↔B→C	YES	9.956×10⁻⁷	2.575×10⁻⁷	9.988×10⁻²	2.342×10⁻¹¹

Table S4. The calculated kinetic parameters and initial maximum fluxes for the system with Aliquat 336 as a carrier.

Ion	No.	Scheme	$V = f(t)$	k_1 [1/cm]	k_{-1} [1/cm]	k_2 [1/cm]	J_M [mol/cm²s]
**Cd(II)**	1	A→B	NO	1.410×10⁻⁵			3.476×10⁻¹⁰
	1a	A→B	YES	1.365×10⁻⁵			3.347×10⁻¹⁰
	2	A↔B	YES	1.364×10⁻⁵	3.661×10⁻¹⁵		3.345×10⁻¹⁰
	3	A→B→C	YES	**1.516×10⁻⁵**		**8.377×10⁻⁵**	**3.718×10⁻¹⁰**
	4	A↔B→C	YES	1.516×10⁻⁵	9.358×10⁻¹⁵	8.373×10⁻⁵	3.718×10⁻¹⁰
**Zn(II)**	1	A→B	NO	6.263×10⁻⁶			1.512×10⁻¹⁰
	1a	A→B	NO	1.365×10⁻⁵			3.347×10⁻¹⁰
	2	A↔B	NO	6.133×10⁻⁶	5.749×10⁻¹⁴		1.473×10⁻¹⁰
	3	A→B→C	YES	6.993×10⁻⁶		4.524×10⁻⁵	1.680×10⁻¹⁰
	4	A↔B→C	YES	**6.743×10⁻⁶**	**3.573×10⁻⁹**	**4.527×10⁻⁵**	**1.620×10⁻¹⁰**
**Pb(II)**	1	A→B	NO	1.365×10⁻⁶			3.340×10⁻¹¹
	1a	A→B	YES	1.341×10⁻⁶			3.265×10⁻¹¹
	2	A↔B	YES	1.341×10⁻⁶	8.388×10⁻¹³		3.265×10⁻¹¹
	3	A→B→C	YES	1.381×10⁻⁶		1.071×10⁻⁴	3.363×10⁻¹¹
	4	A↔B→C	YES	**1.406×10⁻⁶**	**3.701×10⁻⁹**	**7.518×10⁻⁵**	**3.424×10⁻¹¹**

Table S5. The calculated kinetic parameters and initial maximum fluxes for the system with Cyphos IL 101 as a carrier.

Ion	No.	Scheme	$V = f(t)$	k_1 [1/cm]	k_{-1} [1/cm]	k_2 [1/cm]	J_M [mol/cm²s]
Cd(II)	1	A→B	NO	1.301×10⁻⁵			3.206×10⁻¹⁰
	1a	A→B	NO	1.251×10⁻⁵			3.067×10⁻¹⁰
	2	A↔B	YES	1.251×10⁻⁵	6.881×10⁻¹³		3.067×10⁻¹⁰
	3	A→B→C	YES	**1.322×10⁻⁵**		**1.605×10⁻⁴**	**3.240×10⁻¹⁰**
	4	A↔B→C	YES	1.322×10⁻⁵	3.125×10⁻¹⁵	1.605×10⁻⁴	3.241×10⁻¹⁰
Zn(II)	1	A→B	NO	9.179×10⁻⁶			2.254×10⁻¹⁰
	1a	A→B	NO	8.965×10⁻⁶			2.190×10⁻¹⁰
	2	A↔B	NO	8.965×10⁻⁶	2.171×10⁻¹³		2.190×10⁻¹⁰
	3	A→B→C	NO	9.444×10⁻⁶		1.313×10⁻⁴	2.307×10⁻¹⁰
	4	A↔B→C	YES	**9.145×10⁻⁶**	**5.183×10⁻⁹**	**1.431×10⁻⁴**	**2.234×10⁻¹⁰**
Pb(II)	1	A→B	NO	3.077×10⁻⁶			7.189×10⁻¹¹
	1a	A→B	NO	3.011×10⁻⁶			7.000×10⁻¹¹
	2	A↔B	NO	3.011×10⁻⁶	1.032×10⁻¹³		7.000×10⁻¹¹
	3	A→B→C	YES	**3.084×10⁻⁶**		**1.762×10⁻⁴**	**7.170×10⁻¹¹**
	4	A↔B→C	YES	3.084×10⁻⁶	2.668×10⁻¹⁰	1.766×10⁻⁴	7.168×10⁻¹¹
Cu(II)	1	A→B	NO	2.917×10⁻⁸			7.139×10⁻¹³
	1a	A→B	NO	2.840×10⁻⁸			6.916×10⁻¹³
	2	A↔B	YES	**4.559×10⁻⁸**	**4.029×10⁻⁶**		**1.110×10⁻¹²**
	3	A→B→C	NO	2.840×10⁻⁸		39.87	6.916×10⁻¹³
	4	A↔B→C	NO	2.975×10⁻⁸	9.148×10⁻⁵	37.90	6.917×10⁻¹³

Table S6. The calculated kinetic parameters and initial maximum fluxes for the system with D2EHPA as a carrier.

Ion	No.	Scheme	$V = f(t)$	k_1 [1/cm]	k_{-1} [1/cm]	k_2 [1/cm]	J_M [mol/cm²s]
Cd(II)	1	A→B	YES	2.006×10⁻⁶			4.684×10⁻¹¹
	1a	A→B	YES	1.957×10⁻⁶			4.548×10⁻¹¹
	2	A↔B	YES	1.971×10⁻⁶	4.014×10⁻⁸		4.579×10⁻¹¹
	3	A→B→C	YES	**2.047×10⁻⁶**		**7.760×10⁻⁵**	**4.757×10⁻¹¹**
	4	A↔B→C	YES	2.002×10⁻⁶	4.421×10⁻¹⁶	1.482×10⁻⁴	4.652×10⁻¹¹
Zn(II)	1	A→B	NO	1.508×10⁻⁵			3.514×10⁻¹⁰
	1a	A→B	NO	1.472×10⁻⁵			3.413×10⁻¹⁰
	2	A↔B	NO	1.475×10⁻⁵	1.668×10⁻⁸		3.421×10⁻¹⁰
	3	A→B→C	YES	**1.521×10⁻⁵**		**3.570×10⁻⁴**	**3.527×10⁻¹⁰**
	4	A↔B→C	YES	1.514×10⁻⁵	1.314×10⁻⁹	3.634×10⁻⁴	3.512×10⁻¹⁰
Pb(II)	1	A→B	NO	1.178×10⁻⁵			2.792×10⁻¹⁰
	1a	A→B	YES	1.165×10⁻⁵			2.747×10⁻¹⁰
	2	A↔B	YES	1.165×10⁻⁵	3.608×10⁻¹³		2.747×10⁻¹⁰
	3	A→B→C	NO	1.222×10⁻⁵		2.293×10⁻⁴	2.881×10⁻¹⁰
	4	A↔B→C	YES	**1.197×10⁻⁵**	**9.125×10⁻⁹**	**2.235×10⁻⁴**	**2.822×10⁻¹⁰**
Cu(II)	1	A→B	YES	8.925×10⁻⁷			2.180×10⁻¹¹
	1a	A→B	NO	8.729×10⁻⁷			2.121×10⁻¹¹
	2	A↔B	YES	**9.978×10⁻⁷**	**9.512×10⁻⁷**		**2.425×10⁻¹¹**
	3	A→B→C	NO	8.729×10⁻⁷		9.983×10⁻²	2.121×10⁻¹¹
	4	A↔B→C	NO	8.731×10⁻⁷	2.355×10⁻⁵	10.32	2.122×10⁻¹¹

Table S7. The calculated kinetic parameters and initial maximum fluxes for the system with reactive ionic liquid (RILC8_Br) as a carrier

Ion	No.	Scheme	V = f(t)	k_1 [1/cm]	k_{-1} [1/cm]	k$_2$ [1/cm]	J_M [mol/cm²s]
Cd(II)	1	A→B	NO	3.197×10^{-6}			7.499×10^{-11}
	1a	A→B	NO	3.111×10^{-6}			7.260×10^{-11}
	2	A↔B	YES	**3.938×10^{-6}**	**1.565×10^{-6}**		**9.190×10^{-11}**
	3	A→B→C	NO	3.111×10^{-6}		18.03	7.260×10^{-11}
	4	A↔B→C	NO	3.111×10^{-6}	2.264×10^{-4}	98.93	7.260×10^{-11}
Zn(II)	1	A→B	NO	5.914×10^{-7}			1.386×10^{-11}
	1a	A→B	NO	5.767×10^{-7}			1.345×10^{-11}
	2	A↔B	YES	**1.035×10^{-6}**	**4.822×10^{-6}**		**2.389×10^{-11}**
	3	A→B→C	NO	5.768×10^{-7}		25.47	1.345×10^{-11}
	4	A↔B→C	NO	5.770×10^{-7}	4.157×10^{-5}	17.04	1.346×10^{-11}
Pb(II)	1	A→B	YES	4.588×10^{-7}			1.076×10^{-11}
	1a	A→B	YES	4.498×10^{-7}			1.049×10^{-11}
	2	A↔B	YES	**5.762×10^{-7}**	**1.876×10^{-6}**		**1.344×10^{-11}**
	3	A→B→C	YES	4.498×10^{-7}		59.27	1.049×10^{-11}
	4	A↔B→C	YES	4.500×10^{-7}	1.056×10^{-5}	4.436	1.050×10^{-11}

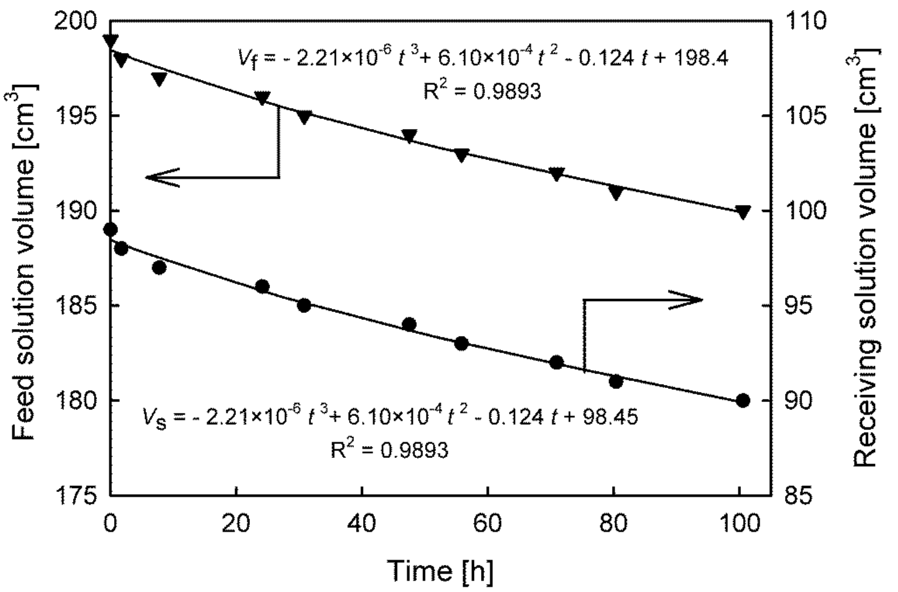

Figure S1. The 3rd degree polynomial fitting to the experimental results for system with D2EHPA as a carrier.

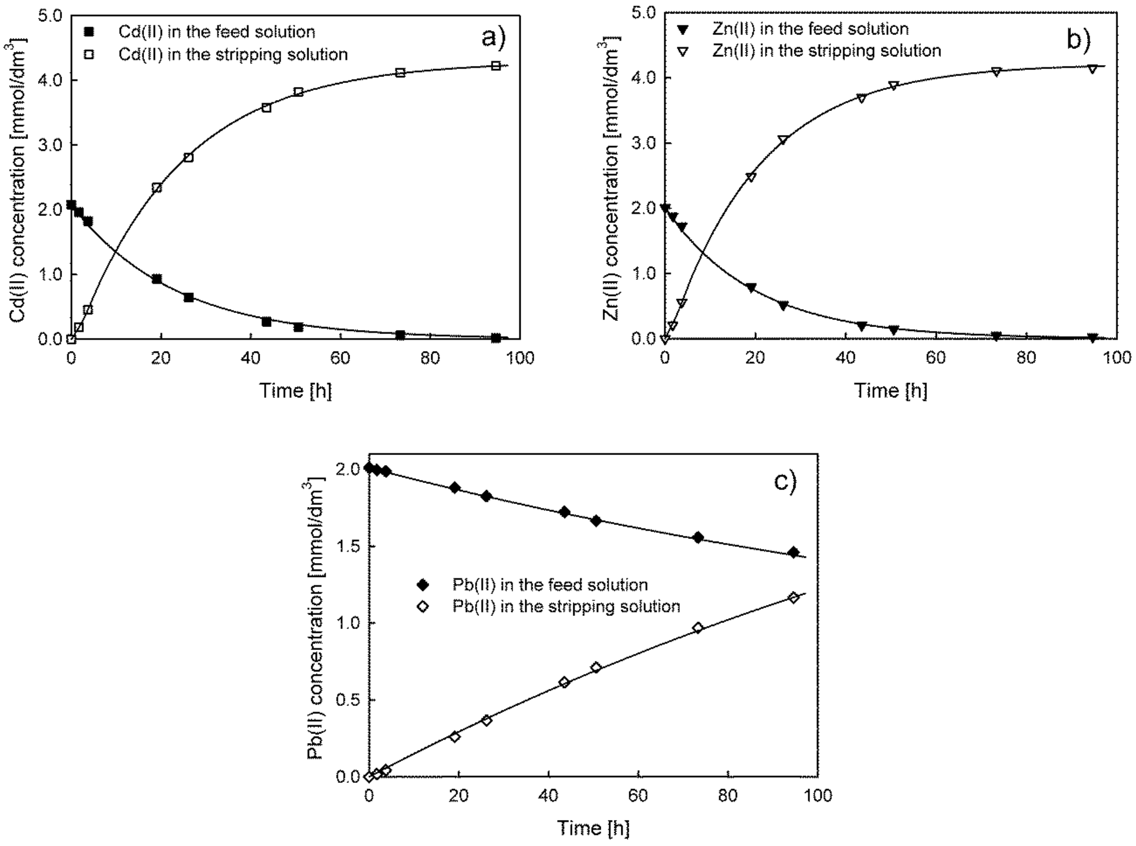

Figure S2. The best fitted models for predicting Cd(II) (a), Zn(II) (b), and Pb(II) (c) ions transport through PIM with TOPO as a carrier.

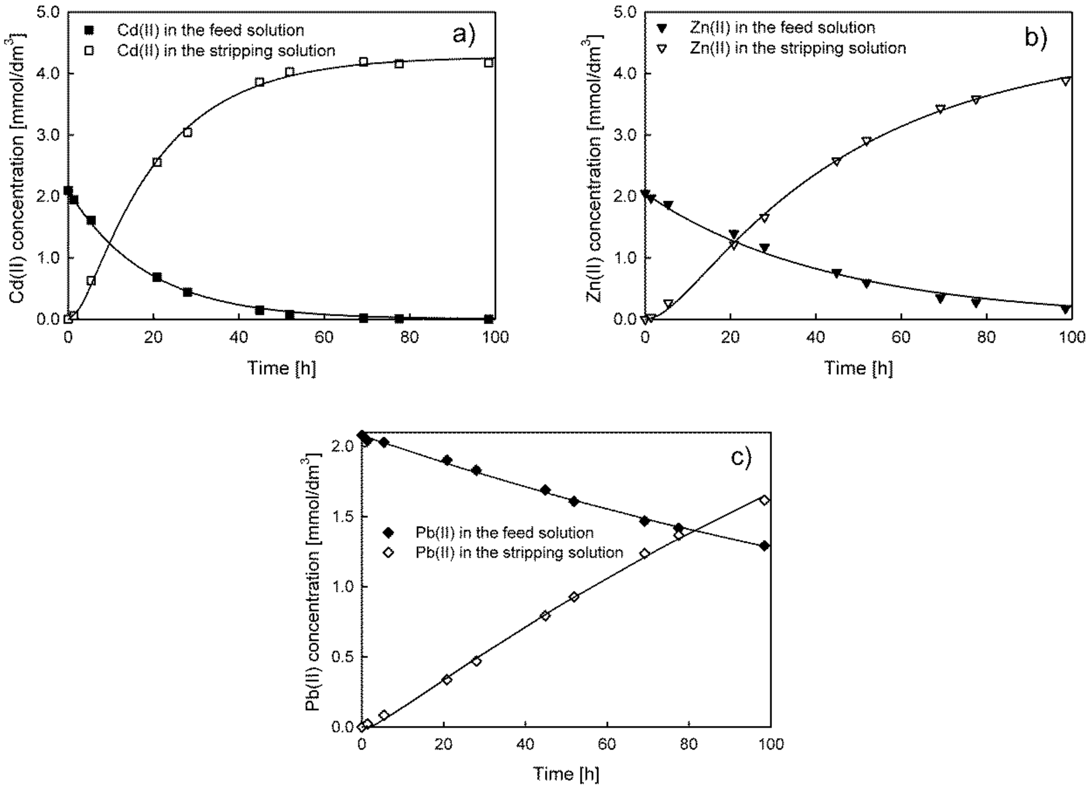

Figure S3. The best fitted models for predicting Cd(II) (a), Zn(II) (b), and Pb(II) (c) ions transport through PIM with Aliquat 336 as a carrier.

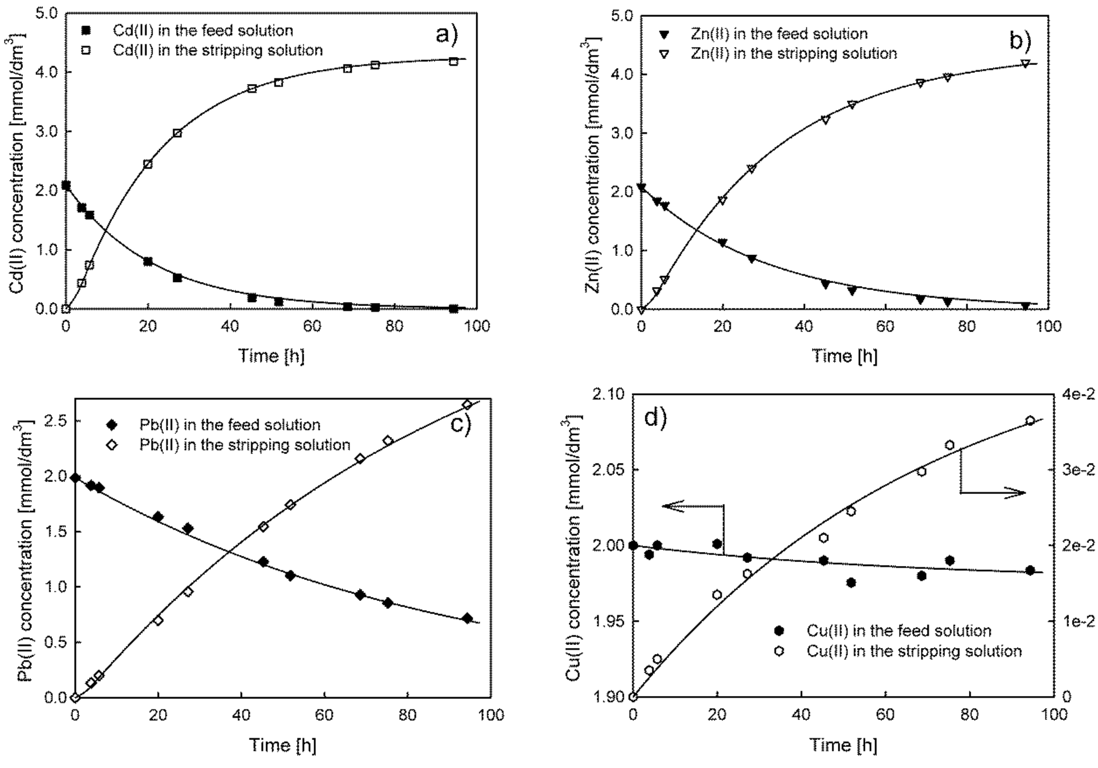

Figure S4. The best fitted models for predicting Cd(II) (a), Zn(II) (b), Pb(II) (c), and Cu(II) (d) ions transport through PIM with Cyphos IL 101 as a carrier.

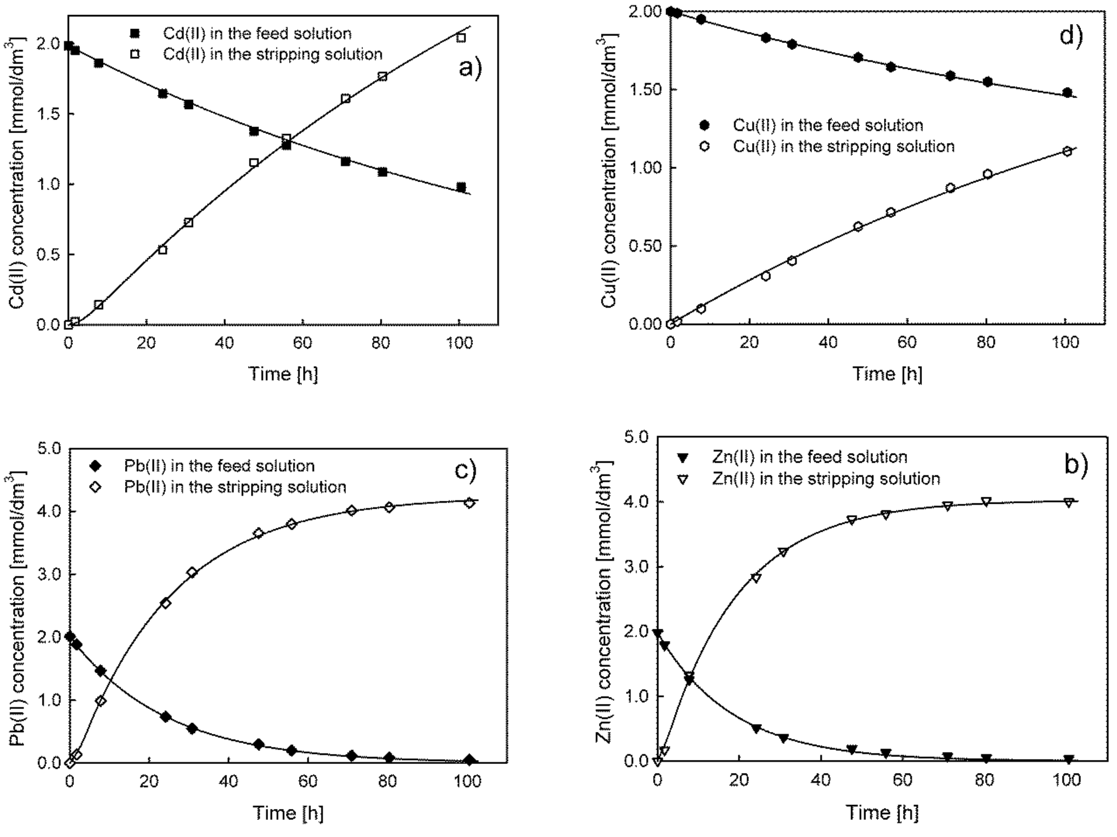

Figure S5. The best fitted models for predicting Cd(II) (a), Zn(II) (b), Pb(II) (c), and Cu(II) (d) ions transport through PIM with D2EHPA as a carrier.

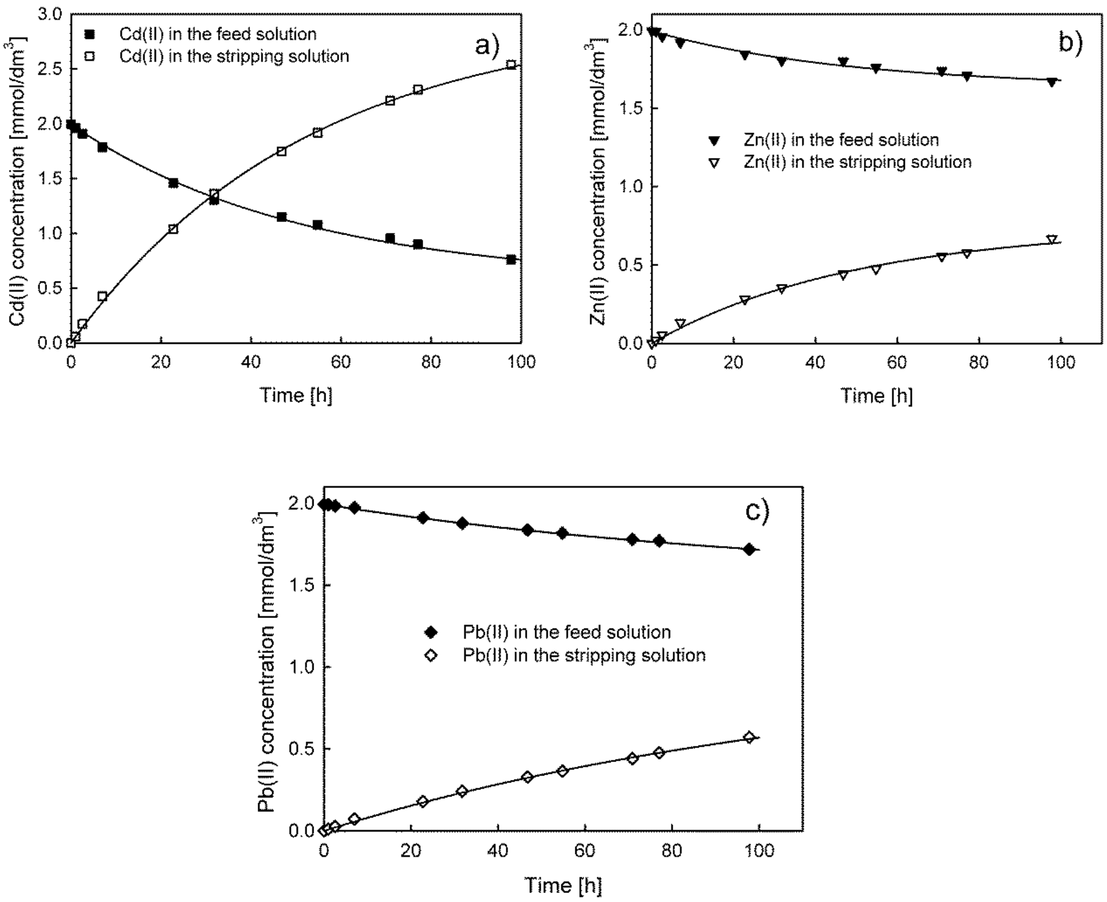

Figure S6. The best fitted models for predicting Cd(II) (a), Zn(II) (b), and Pb(II) (c) ions transport through PIM with a reactive ionic liquid (RILC8_Br) as a carrier.
